# Local Renyi entropic profiles of DNA sequences

**DOI:** 10.1186/1471-2105-8-393

**Published:** 2007-10-16

**Authors:** Susana Vinga, Jonas S Almeida

**Affiliations:** 1 Instituto de Engenharia de Sistemas e Computadores: Investigação e Desenvolvimento (INESC-ID), R. Alves Redol 9, 1000-029 Lisboa, Portugal; 2 Departamento de Bioestatística e Informática, Faculdade de Ciências Médicas – Universidade Nova de Lisboa (FCM/UNL), Campo dos Mártires da Pátria 130, 1169-056 Lisboa, Portugal; 3 Dept Biostatistics and Applied Mathematics, Univ. Texas MDAnderson Cancer Center – unit 447, 1515 Holcombe Blvd, Houston TX 77030-4009, USA; 4 Biomathematics Group, Instituto de Tecnologia Química e Biológica – Universidade Nova de Lisboa (ITQB/UNL), R. Qta. Grande 6, 2780-156 Oeiras, Portugal

## Abstract

**Background:**

In a recent report the authors presented a new measure of continuous entropy for DNA sequences, which allows the estimation of their randomness level. The definition therein explored was based on the Rényi entropy of probability density estimation (pdf) using the Parzen's window method and applied to Chaos Game Representation/Universal Sequence Maps (CGR/USM). Subsequent work proposed a fractal pdf kernel as a more exact solution for the iterated map representation. This report extends the concepts of continuous entropy by defining DNA sequence entropic profiles using the new pdf estimations to refine the density estimation of motifs.

**Results:**

The new methodology enables two results. On the one hand it shows that the entropic profiles are directly related with the statistical significance of motifs, allowing the study of under and over-representation of segments. On the other hand, by spanning the parameters of the kernel function it is possible to extract important information about the scale of each conserved DNA region. The computational applications, developed in Matlab m-code, the corresponding binary executables and additional material and examples are made publicly available at .

**Conclusion:**

The ability to detect local conservation from a scale-independent representation of symbolic sequences is particularly relevant for biological applications where conserved motifs occur in multiple, overlapping scales, with significant future applications in the recognition of foreign genomic material and inference of motif structures.

## Background

Biological sequences are the ultimate support for the description of Biological Systems. In particular, key aspects of sequence analysis are known to play a role in integrated analysis of regulatory networks: for example in motif searching and inference.

Over the last decades and more recently due to the development of a considerable number of whole genome sequencing projects, several efforts have been made to mathematically model DNA sequences. In particular from the statistical side, the use of Markov based models [1] has widespread and proven to be effective in tackling the problem of data mining of biological sequences, through variable length Markov chains [2,3], interpolated Markov models [4], fractal prediction machines [5] for symbolic time series based on Chaos Game Representations [6], to name just a few. Other algorithmic approaches based on the computational side have also proven to be useful [7]. All this effort allowed establishing important relations between the results obtained (computationally and statistically) with real biologically significant findings. From these models developed for DNA, it is now apparent that each genome has pervasive [8] motif and compositional characteristics in terms of the frequencies of its constitutive *L*-tuples or *L*-length motifs, which gave rise to the genomic signature concept [9]. This fact can be directly employed for horizontal transfer detection and characterization, coding vs. non-coding discrimination [8,10], study and compare DNA through the use of composition profiles [11] and spectra [12] and other applications partly reviewed in [13].

In this regard and more specifically, an important statistical problem in bioinformatics that emerged is the evaluation of the number of repetitions occurring in biological sequences. More generally, they can occur in distinct hierarchical levels, from single symbols [14] to genes. In fact, in a recent paper, the number of gene repetitions was shown to be a key aspect of gene expression and phenotype [15]. Apparently theses repetitions, not only at nucleotide level, might play a key role in genome organization and functionality of networks. The notions of repetitions, entropy and correlation in DNA are unquestionably connected [16-18] and references therein – the degree of predictability of a sequence, which is closely related with its internal repetition and compression, can be measured by its entropy. The major importance of this research has provided evidence that is already too vast to fully account for. In particular, the relation between motif over- or under-representation is usually related with their biological function. This creates the need for an efficient method to analyze, for different parameters sets, the degree or scale of each DNA region.

In a recent report [19], the authors defined a new continuous measure of DNA entropy, based on non-parametric density estimation applied to Chaos Game Representation (CGR) and Universal Sequence Maps (USM) within the Rényi theory. The idea therein explored was that there is a close relationship between the statistics of the sequences, given by their constitutive motifs, and their entropy, measured under information theory methodologies. In that report the Rényi entropy was estimated in a global approach, and the measures obtained were compared with random sequences by Monte Carlo simulation. Although the main concepts were then introduced, that report was incomplete in the sense that just a global analysis was conducted. Specifically, no exploration of local patterns and fine tuned neighboring analysis was conducted, which is finally allowed by the present work, with the introduction of the concept of the *Entropic Profile *(EP).

Entropic profiles were defined previously but in a different context and scope: they were estimated using the histograms of the *L*-mer or *L*-tuple frequencies in DNA [20]. In that report the authors could discriminate between random and natural DNA sequences using the Shannon entropies of the histograms obtained from the CGR for different resolutions or oligomer lengths. Although the same name was used, that previous endeavor focused on a global perspective of sequence entropy [19] whereas this report proposes and investigates a local entropy formulation instead. In fact, the results obtained by Oliver and colleagues are global features for each DNA sequence, different from the present proposal of local based information per position/symbol. Another type of sequence profile also explored was based on linguistic complexity [21] and low entropy DNA zones [22].

In the present report the definition of entropic profile arises from the direct estimation of a local density, derived from the Parzen's window method described before. In our last report this estimation allowed the calculation of a global entropy measure, according to the Rényi definition. This report describes the next logical step of exploring complementary methods to access local information as to identify the location and composition of the conserved sequence which existence might have been anticipated from the global measures of entropy. The rationale is to have a function that assesses, for each position in the sequence (illustrated here for DNA), the information content of *L*-tuple suffixes directly from the density kernel function estimate. Such a solution should enable the scale-independent extraction of motifs without the need to identify complex state automata for unit succession.

In addition to our preceding report on Rényi entropy for global characterization of sequences, the study reported here also builds on the identification of a kernel function that produces a more accurate density estimation in CGR/USM projections of symbolic sequences [23]. The more conventional use of symmetrical functions as we did with a Gaussian Parzen kernel produces a rough fit to the characteristically fractal nature of iterative map projections. That approximation did suffice for assessment of global entropy [19] but it is not refined enough for the intended density estimation resolved locally at the sequence unit level.

Future applications of the methodologies here proposed might include motif inference and extraction, providing tools for the construction and inference of generalized sequence models for whole genomes.

## Results and Discussion

This section presents some entropic profiles calculated for the DNA sequences described below. The relation between this values and former results is also investigated. Additionally, the influence of the parameters on the profiles is discussed.

### DNA sequence dataset description

For sake of clarity this report uses the same dataset previously studied [19], thus allowing a comparison of results, in the continuity of the former proposal. In particular, the results for a subset of those sequences with known present motifs will be shown and extensively studied. In order to further test the estimation of the profiles to more challenging datasets, the analysis of whole genomes is also included. More specifically, the detection of Chi sequences in *Escherichia coli *and *Haemophilus influenzae *will be assessed. These genomes have been extensively analyzed after the completion of its DNA sequencing projects, thus constituting an excellent dataset to test new procedures. In particular, several important motifs have been studied elsewhere and can be compared directly with the proposed method. The following Table 1 recalls the DNA sequences examined.

**Table 1:** DNA sequence dataset used in this report.

***Name***	***Sequence description***	***Length [bp]***
m3	random with inserted motif *L *= 3 'ATC'	2000
m4	random with inserted motif *L *= 4 'ATCG'	2000
m5	random with inserted motif *L *= 5 'ATCGA'	2000
Es	experimental promoter regions of *B. subtilis*	2000
Ec	*Escherichia coli *K12, complete genome [GenBank:NC_000913]	4639675
Hi	*Haemophilus influenzae *Rd KW20, complete genome [GenBank:NC_000907]	1830138

The artificial sequences m3, m4 and m5 are obtained by generating random DNA (with symbol emission probabilities *p*_A _= *p*_T _= *p*_C _= *p*_G _= 0.25) and subsequently implanting the motifs described (respectively 'ATC', 'ATCG' and 'ATCGA') in specific positions. The sequence Es corresponds to the concatenation of real DNA from 20 promoter regions of *Bacillus subtilis *[45, 46], with known consensus structured motif *TTGACA-(space)-TATAAT *with at most one point mutation or substitution. The sequences Ec and Hi are the complete genomes of *Escherichia coli *and *Haemophilus influenzae *extracted from NCBI GenBank.

All the datasets and additional information are available in the webpage referred to above.

### Entropic profiles and parameters optimization

The tests consisted on calculating the entropic profiles (EP) for different combination of parameters *L *and *φ *and check for particular features. The use of artificial DNA allows the accurate study of the impact of the parameters on the profiles obtained. The results can be directly obtained by using the deduced formulae of Equations 5 for $\hat{f}$_*L, φ *_(*x*_*i*_) and their corresponding normalized values $\hat{g}$_*L, φ *_(*x*_*i*_) (Eq.3), after specifying the parameters (see Methods and online software).

**Figure 1:**
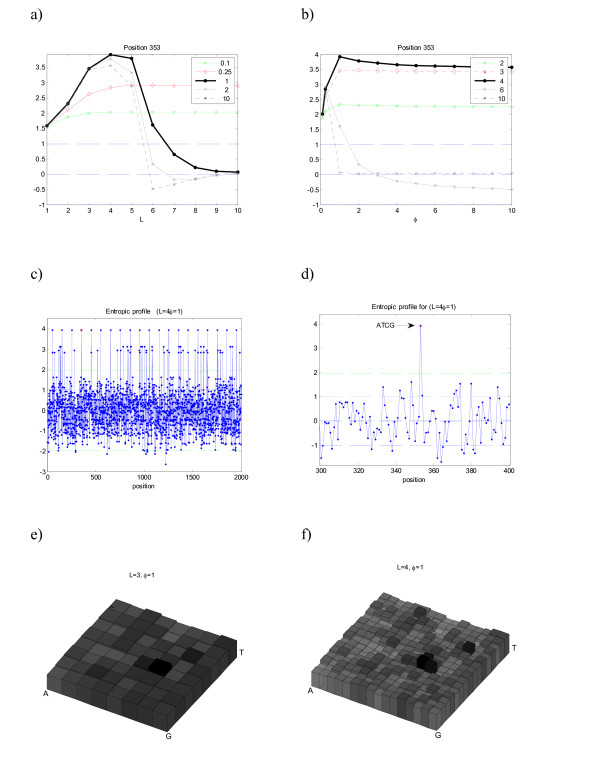
**Entropic profile (EP) for sequence m4**. Artificial DNA sequence with implanted motif "ATCG" in positions [50*i*...53*i*+100], *i *= 0, ..., 19 (see Table 1). Several parameter combination *L *and *φ *are presented, and the corresponding EP values are plotted a) as a function of *L *for several *φ *and b) as a function of *φ *(for the same *L *values). The maximum values of normalized estimations *g *vary along the positions. In this example, position 353 corresponds to the last symbol of one (randomly chosen) occurrence of motif ATCG, and its EP attains a maximum value for *L *= 4 and *φ *= 1, with more than 3.5 standard deviations from the mean densities (EP _*θ*_(353) = 3.8). c) and d) The complete profile for these parameter values, showing the peaks on the implanted suffix ATCG. The most representative parameter values are plotted. e) and f) The CGR densities obtained from the profiles using the fractal kernel described.

The results presented in this section are focused on the analysis of specific positions, known to be important and/or contain statistical significant motifs as suffixes. For example, Figure 1 represents the profiles obtained for the sequence m4 with the motif 'ATCG' implanted. This motif was implanted *n *= 20 times at equally spaced positions p = 50+*i*100, *i *= 1, ..., 20 (see details in [19]). By studying one of the positions where this suffix ends (as an illustrative example *p *= 353 was chosen), one immediately assesses for which combination of parameters *L *and *φ *the maximum values of the profiles is obtained. In this case this maximum is achieved with *L *= 4 and *φ *approximately of 1 (one might further search this parameter space continuously in order to optimize *φ *but this is not pertinent in this explanatory step).

As seen from the Figure 1a) and 1b), there are parameter combinations for which that particular position/suffix is highlighted, with normalized density values way above alternative choices. It was not by chance that the maximum was attained at *L *= 4, since this is precisely the length of the suffix highly repeated, so *L*_max _≥ 4 was expected to be a local maximum of EP.

In the other panels of Figure 1 the entropic profile for the complete sequence is plotted, using the parameters previously optimized for the chosen position (*p *= 353). These plots allow the overview of all the sequence using local information obtained for a specific putative important suffix and, in fact, using this combination of parameters one immediately recovers all the positions where the known motif appears, which are simply the peaks on the graph. Panel d) shows a detail of the EP (from position 300 to 400), clearly illustrating the position where the implanted motif "ATCG" ends, with a density local maximum around EP(353) = 3.9. The expected number of counts under a first-order Markov Chain model would be 10.7 (p-value = 0.0027, z-score = 2.78).

In Figure 1e) and 1f) is also shown the corresponding density estimations on the CGR map for two distinct parameter sets. Comparatively with the Gaussian function this kernel is better adjusted to the CGR square-based geometry and presents a more clear-cut profile, as expected. The darker squares correspond precisely to the implanted motif sub-quadrants.

The following figures present the same results obtained with the other datasets under study.

**Figure 2:**
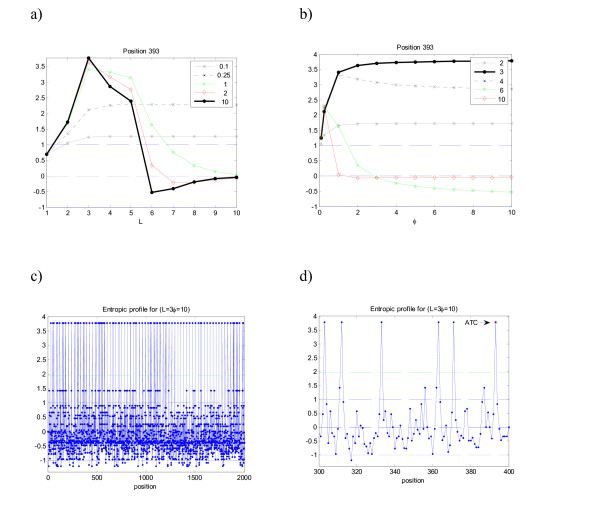
**Entropic profile (EP) for sequence m3**. Artificial DNA sequence with implanted motif "ATC" in positions [30*i*+1...30*i*+3], *i *= 1, ..., 66 (see Table 1). Same analysis conducted. See legend of Figure 1.

In Figure 2 the same pattern occurs, with maxima EP(393) = 3.8, obtained for *L *= 3, again the implanted motif length. It should be mentioned that occasionally, for some positions where the motif "ATC" appears, the maxima occurs for a value *L *> 3. This can also happen and simply means that longer, non-implanted motifs appeared more often that would be expected by chance – in this case "ATC" is embedded in a longer significant motif, i.e. is contained in a longer string with potential significance. Interestingly, when plotting all the EP for the sequence using *L *= 3, one obtains additional, non-implanted motifs, which occurred just by chance – extra peaks with non-equal spacing in Figure 2c) and 2d). In fact, the probability of one specific motif of length 3 (under a null model of symbol equiprobability) is 4^-3^, which implies, for a sequence of 2000, that the expected number of counts is roughly equal to 31. This simply means that the motif already existed in the random sequence m3 before the implantation took place. The detail graph – Fig. 2d) – shows precisely these "extra" appearances. If one uses a first-order Markov chain model as previously the expected number of counts becomes 60.08 (p-value = 2.8E-10, z-score = 6.2).

**Figure 3:**
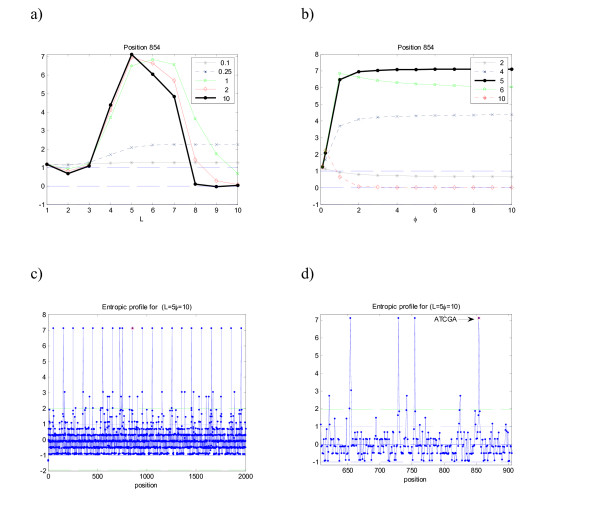
**Entropic profile (EP) for sequence m5**. Artificial DNA sequence with implanted motif "ATCGA" in positions [50+100*i*...54+100*i*], *i *= 0, ..., 19 (see Table 1). Position analysis for sequence m5, analogous to those conducted previously. See legend of Figure 1.

A similar interpretation can be made regarding sequence m5: the positions where the suffix "ATCGA" appears have maximal values $\hat{g}$ (*x*) for *L *= 5, although with high values in the range *L *= 4 to *L *= 7, which indicate nested significant motifs. The entropic profile for the complete 2000 base-sequence shows the maxima of the equally spaced motif (see Fig. 3), where it is noticeable an extra peak that corresponds to a previously existing motif ATCGA (ending at position 729).

By spanning the parameters space (*L*, *φ*) it is possible to find maximum values for $\hat{g}$ (*x*). For example, in specific positions 854 one finds out that $\hat{g}$ attains a maximum value for memory *L *= 5 and *φ ≥* 10 with EP(854) = 7.1, a high relative value. By using these optima in the EP one obtains a profile that highlights immediately the suffixes where the highly repeated motif appears. Some other maxima appears sometimes (results not shown), but were discovered to correspond to other interpretable extreme values. The expected number of counts for this motif is just 3.07 that, comparing with the observed 21 occurrences, gives a p-value≈0 (z-score = 10.02).

**Figure 4:**
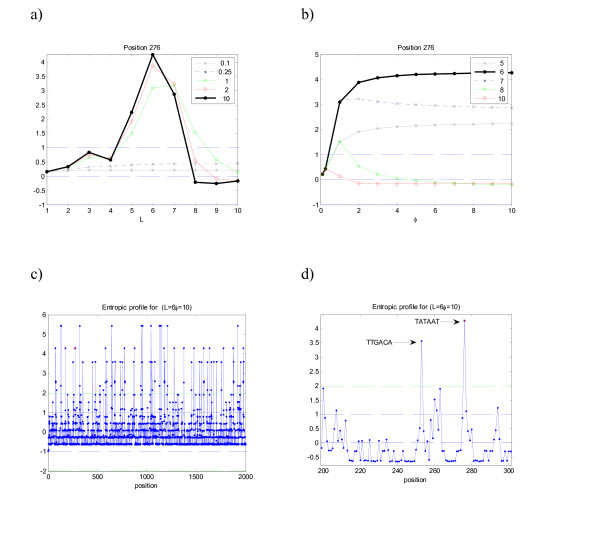
**Entropic profile (EP) for sequence Es of the promoter regions in *B. subtilis***. The peaks in the EP correspond to the structured motif TTGACA-TATAAT. This particular position is well conserved so that the motif is easily detected. Other positions where the motif is degenerated do not exhibit a similar conservation and clear profile. The highest peaks in panel c) correspond to the motif 'AAAAAA', which is repeated more often in the sequence than the previous ones. The overlapping capacity of this motif can partially explain this behavior.

Finally, Figure 4 shows part of the results for the real DNA sequence in the position corresponding to the ending of the TATA box (motif = "TATAAT"). The graph for this position shows precisely that *L *= 6 is an interesting scale to search for. The EP, in contrast to the former ones, does not exhibit a clear trend. In fact, differently to the former sequences, which were artificially generated and presented non-degenerate highly conserved motifs, the real DNA exhibits several point mutations that introduce some "noise" in the estimations. When plotting the complete profile for this sequence and observing one detail it is possible to recover the complete structured motif, known to bind to specific transcription factor binding sites, with values EP(TATAAT) = 4.3 and EP(TTGACA) = 3.6. It should be stressed however, that these results are biased towards the sequence itself: in this particular case, the concatenation of the promoter regions of *B. subtilis *provided a set with conserved motifs, at least to the point where they could be detected by density estimations. Of course, if non-conservation is allowed up to a higher level, the EP becomes noisier and eventually the signal will be lost, hampering the recovering of any significant motif if no pre-processing correction is performed. The analysis based of Markov chains gives for the TATAAT motif an expected number of counts of 1.60 (p-value≈0, z-score = 10.38) and 0.94 for TTGACA (p-value≈0, z-score = 9.54). The most common motif EP(AAAAAA) = 5.4 is highly periodic which explains the peak, although under a Markov chain it is expected to occur 11.67 (p-value = 0.1245, z-score = 1.15).

The two last datasets are constituted by whole genomes from two Gammaproteobacteria: *Escherichia coli *K12 and *Haemophilus influenzae *Rd (see Table 1 for NCBI GenBank accession numbers).

The study of the regions where Chi sequences appear will be analyzed in both genomes. Chi (crossover hotspot instigator) sites are homologous recombinational hotspot octamer sequences which modulate the exonuclease activity of RecBCD. This enzyme is necessary for chromosomal dsDNA repair and integration of exogenous dsDNA, which supports the idea that Chi sites have a biologically functional role [24].

Since Chi motifs are orientation-dependent and strand-specific, the sequence to be analyzed should be previously processed to comply with this property. This means that one should extract the whole genome and use the DNA sequence from the origin of replication up to the terminus plus the reverse complementary sequence, since chromosome replication in bacteria start from one replication origin (oriC) and proceeds bi-directionally until the replication forks reach the termination site (terC). These pre-processed genomes will conform the 5'->3' direction of replication and therefore will be used throughout the analysis. The oriC and terC positions (referred to the NCBI GenBank database) have been estimated based on experimental data and asymmetric properties [25] and are specified in Table 2.

**Table 2:** Description of Chi sites in E. coli and H. influenzae genomes.

***Genome***	***Chi sequence***	***Nr. occurrences***
*E. coli*	5'-GCTGGTGG-3'	761
oriC – 3,923,500 (bp)		
terC – 1,588,800 (bp)		
*H. influenzae*	5'-G**G**TGGTGG-3'	77
oriC – 603,000 (bp)	5'-G**C**TGGTGG-3'	56
terC – 1,518,000 (bp)	5'-G**T**TGGTGG-3'	63
5'-G**N**TGGTGG-3'	5'-G**A**TGGTGG-3'	28
5'-G**S**TGGAGG-3'	5'-G**G**TGGAGG-3'	11
	5'-G**C**TGGAGG-3'	7

The number of occurrences of Chi motifs in the genomes shows that they are overrepresented in *E. coli *(761 occurrences) but not in *H. influenzae *(maximum of 77 occurrences).

Chi sequences (see Table 2) are statistically overrepresented in the genome of *E. coli *(5'-GCTGGTGG-3'), appearing more often than would be expected by chance whereas in *H. influenzae *(5'-GNTGGTGG-3' and 5'-GSTGGAGG-3' show Chi activity) they are known to be less frequent and less conserved. This makes for two different datasets with distinct features that involve a different degree of difficulty to detect these regions.

The study of Chi sites have been subject to many analyses and therefore constitute an excellent test dataset to assess the strength of the entropic profile approach to detect these motifs. In particular several recent papers have assessed its statistical significance using Markov models [1], analyzing the 8-tuple frequency for the whole genome of *E. coli *[26] and also comparing Chi site conservation in both organisms [24].

The expected number of an 8-tuple in *E. coli *and *H. influenzae *using a Markov model of order 0 (only nucleotide abundance is taken into account) is respectively 70.796 and 27.926. One immediately sees that in *E. coli *this motif is highly represented whereas in *H. influenzae *this fact is less evident.

Interestingly, when analyzing whole genomes, several motifs appear with p-values near 0, i.e. they occur in exceptionally high number when considering a Markov chain model. This fact does not allow their accurate comparison and is a major drawback of using solely the p-values to assess the statistical significance and correctly compare and order the relative importance of these motifs. Therefore, as explained in the Methods, the normalized z-scores are also reported for clarity.

For example, using a first order Markov Chain model the expected number of counts for the chi-sequence in *E. coli *and *H. influenza *is 85.06 and 12.34 respectively. Although this motif has a p-value≈0 for both sequences, the corresponding z-score of 73.37 and 12.43 respectively puts it in different ranks among all motifs of the same length.

**Figure 5:**
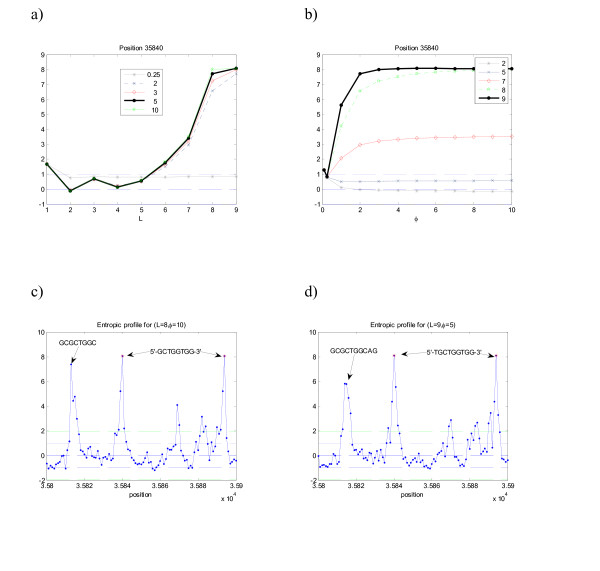
**Entropic profile (EP) for sequence Ec – complete genome of *E. coli***. a) and b) Analysis of position 35840 (from the beginning of replication). c) and d) Detail for positions 35800 to 35900. The peaks in the EP correspond to the Chi sequence motif 5'-GCTGGTGG-3'. This particular position is well conserved so the motif is easily detected.

When analyzing one (random) position where Chi sequence ends in *E. coli *(exactly in the same way as the previous analysis) the following profiles are obtained (Fig. 5). The position p = 35840 shows that the maximum EP values are obtained for parameters (*L *= 8, *φ *= 10) and (*L *= 9, *φ *= 5), for which the profiles attain similar values of EP = 8.04 and EP = 8.08 respectively. For *L *= 7 the motif also appears relevant. The complete profiles for that region are plotted in the panels c) and d), showing striking and evident peaks at the positions where Chi sequences end. The other local maximum corresponds to a chi-related sequence (GCGCTGGC), which in fact shares the 5-mer GCTGG. Indeed, the family containing the trimer CTG, often within the pentamer GCTGG, is very frequent in this genome [27], all with p-values≈0 and highest scoring ranks

**Figure 6:**
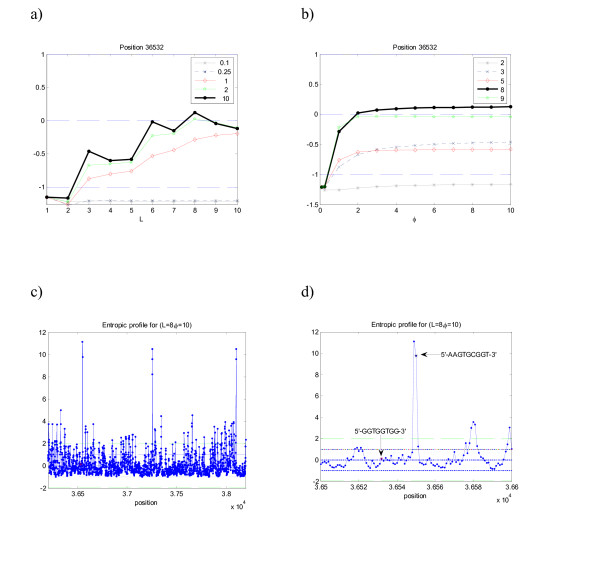
**Entropic profile (EP) for sequence Hi – complete genome of *H. influenzae***. a) and b) Analysis of position 36532 (from the beginning of replication). c) and d) Detail for the EP for positions 36200 to 38200 and 36500 to 36600. The highest peaks in the EP correspond to uptake signal sequences (USS+) 5'-AAGTGCCGGT-3', its reverse complement (USS-) 5'-ACCGCACTT-3' and related motifs, such as AGTGCGGT and AAGTGCGG. The Chi sites are not particularly well conserved neither overexpressed [24] and therefore are not easily detected with this approach.

When analyzing the genome of *H. influenzae *and studying one particular position where motif 5'-GGTGGTGG-3' ends (in the example, p = 36532), the following Figure 6 is obtained.

From Figure 6a) is it possible to see that the maximum EP = 0.1252 is obtained for parameters (*L *= 8, *φ *= 10), a relatively low value when compared with the previous examples so far. Interestingly other peaks exhibit a period of 3 (*L *= 3 and *L *= 6) – the motif TGGTGG repeats every 3 and 6 bases and therefore that property is patent in the graph (Figure 6a) through the appearance of this local maxima every 3 bases. When using the above parameters to plot the entire profile one immediately sees that other positions of extremely high significance appear. This is the case of the 8-tuple motifs AAGTGCGG and AGTGCGGT, which corresponds to EP(36549) = 11.1281, p-value≈0, z-score = 174.80, and EP(36550) = 9.7819, p-value≈0, z-score = 186.20, marked in Figure 6d). These motifs appear 867 and 770 times in the genome, which makes them the most common 8-tuples, along with CCGCACTT (820 times; EP = 10.4869, p-value≈0, z-score = 184.47), ACCGCACT (755 times; EP = 9.5784, p-value≈0, z-score = 210.81) and AAAGTGCG (699 times; EP = 8.8696, p-value≈0, z-score = 97.35), using the same parameters.

As expected, the Chi sites are not detected solely based on EP maximization. In fact, the motif is not especially over-represented when compared with all the others, so it would be impossible to detect based solely on the raw entropic profiles. Furthermore and evident from the figures, the *H. influenzae *genome has one extremely ubiquitous 9-tuple motif, the extensively studied *uptake signal sequence (USS+) *AAGTGCGGT (appears 740 times) and its inverted complement sequence *(USS-) *ACCGCACTT (731 times) with a total number of 1471 occurrences. Their p-values≈0 and their extremely high z-scores of 293.28 and 329.74, puts them in the first rank positions of exceptionality. Furthermore, all the motifs present among the first 25 highest scoring positions greatly overlap USS sequences [1].

USSs are involved in *natural competence*, which is a genetically controlled form of horizontal gene transfer in some bacterial species, related to their ability to take up DNA from the surrounding environment (reviewed in [28]). This process allows genetic exchange in bacteria, which is the only organism known to actively take up DNA from the environment and recombine it into their own genome [29]. The DNA uptake machinery on the cell surface preferentially binds and takes up fragments containing this specific short sequence. In particular *H. influenzae *is able to take up double-stranded DNA of its own species and closely relatives, facilitated through the recognition of USS, which are indeed over represented in its genome.

One interesting statistical aspect of the USS distribution, besides its extremely over-representation, is that these sequences appear equally partitioned in both strands and are remarkably and significantly evenly spaced around the chromosome [30]. They can be constituted by the 9 bp core referred to but allowing a longer 29 bp consensus. The USS evolutionary origin and function was recently addressed [31] by confronting two models, preference first hypothesis and a molecular drive hypothesis. Nevertheless this issue remains controversial [32].

Through the analysis of *H. influenzae *complete genome conducted above one obtains peaks on the entropic profiles precisely at these ubiquitous motifs, which definitely obscures the retrieval of Chi sequences, whose number of occurrences is not at all comparable with USS frequency.

In fact, the profile obtained for the maximum values (*L*, *φ*) shows that the Chi sequence (with G) attains a maximum entropic density value of 0.12, which is way below the detection level when compared with the value obtained for USS which was equal to EP(AGTGCGGT) = 9.78 and EP(AAGTGCGG) = 11.13. This phenomenon is well understood, and some authors name it "contamination" [1]: the highly overrepresented expressed motif contaminates the calculation of low expressed segments. The program R'MES [33] lists precisely USS motifs and their variants showing this behavior. One idea to assess the statistical significance excluding this bias is to delete, from the original sequence, the regions/positions where this ubiquitous 9-tuple appears [1]. This is approximately comparable to perform exact Markov calculations and therefore can be used to further study the sequence. The obtained values for the transformed sequence were nevertheless very low around EP = 0.16 (results not shown). After investigation what might be happening it was found that other motifs emerged even when USS were all deleted from the genome.

For example, the 8-tuple AAAATTTT (p-value→1, z-score = -10.70) appears with high EP values, along with other motifs constituted by long successions of A's and T's. These long adenine-thymine tracts, previously detected for other organisms such as Yeast [34,35], might have an important role due to their strong DNA bending properties [36]. Although the detection of Chi sites failed, other motifs emerged that have notable biological functions and roles in the cell.

This effort highlight an important possible procedure, to be explored further, that one should plot the motifs hierarchically and delete the influence of more ubiquitous motifs that highly "pollute" the calculations, starting from the most exceptional. In fact, from the profile information we could further envisage an algorithm that automatically extracts putative motifs for each position. This is accomplished by searching the parameters space for which the estimation is maximal for position *i*:

$${\left(L_{\max},\varphi_{\max}\right)}_{i}=\underset{L,\varphi }{\arg\max}{\hat{g}}_{L,\varphi }\left(x_{i}\right)$$

and then use these parameters to retrieve the suffix $m_{i}=s_{i-L_{\max}+1}\cdots s_{i}$.

**Figure 7:**
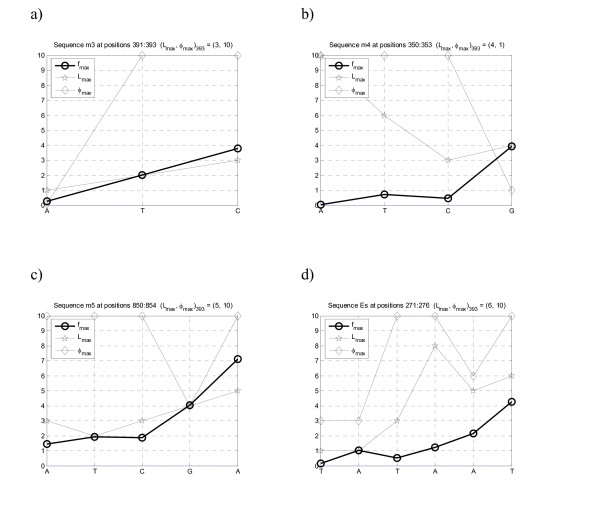
**Conserved motif detection and extraction**. By searching the parameter space (L, *φ*) for a specific position *i *and finding the values ${\left(L_{\max},\varphi_{\max}\right)}_{i}=\underset{L,\varphi }{\arg\max}{\hat{g}}_{L,\varphi }\left(x_{i}\right)$ it is possible to extract the most significant suffix in) the entropic profile context, illustrated here for the first four sequences. Each of the panels corresponds to a different sequence and position where the motif was correctly recovered just by using these maxima: a) m3, b) m4, c) m5 and d) Es (see also Table 1). The profiles for the *L*_max _and *φ*_max _are also shown: apparently one can obtain a non-decreasing function of the positions, which means that previous suffixes are embedded in the implanted motifs.

Using this methodology one obtains precisely the implanted motifs of the previous datasets. As an example, the "TATA"-box referred to before is correctly inferred and also the above mentioned examples with the artificial sequences (Figure 7).

It should be stressed however that this is not the most convenient procedure for motif inference problems since several algorithms already exist that perform these searches very efficiently. Nevertheless is interesting to find that combinatorial and probabilistic methodologies are comparable as the latter come with broader opportunities for theory development albeit leading to advantageous numerical solutions. The observation that there is a close relationship between the overrepresentation, detected by the majority of the algorithms, and the proposed Entropic Profiles with its density and statistical significance measure suggests that it could provide a way of simultaneously finding and statistically classifying the motifs instead of pursuing the two goals separately.

The analysis also showed that the statistical significance z-scores and p-values are unequivocally related with the entropic profiles, since most of the algorithms detected the same motifs. Over-represented motifs exhibit a very low p-value, very near zero, and high z-scores and EP values; common motifs, that appear a median and/or expected number of times, have high p-values and low z-scores, which indicate its non-exceptionality under the Markov chain model considered. These are the motifs that also attain low EP values. The full correspondence between both methods is still under study.

By expressing the density estimation as a function of the suffix counts, one is also allowed to search for under-represented segments, i.e., those whose density is below average. Although not explored in this work, minimum entropic profile values might also play a role in under-represented motifs detection. In fact, rare motifs/substrings are known to correspond to traits/regions with very specific functions in high precision biological processes. The use of unique sub-strings, or UniMarkers, that appear only once in the genome, recently allowed to locate single nucleotide polymorphisms (SNP's) [37,38]. These unique substrings were shown to be clustered close to genes [39]. All these positions can be detected as low-density areas in the CGR and consequently correspond to local minima in their Entropic Profiles. Another example also related with low-density points is related with 6-tuple palindromes. These short sequences, which often correspond to restriction sites, are under-represented in *E. coli *and in the bacteriophage lambda [1,40], thus providing a self-protecting effect. More generally this methodology can be used to find heterogeneous traits in the genome, both related with local under- and over- representation of motifs. This result can indicate the presence of foreign material which can have significant applications in the detection of horizontal transfer [11].

## Conclusion

In this report, Entropic Profiles (EP) were proposed as a novel local information entropy measure for DNA sequences. This function is built on previous work on continuous Rényi quadratic entropy where the Parzen window method was applied to the non-parametric density estimation of the Chaos Game Representation/Universal Sequence Maps (CGR/USM) of a sequence. Subsequently, the estimation was decisively refined to the accuracy that the determination of local entropy requires. This advance, reported elsewhere, introduced a two-parameter fractal-based kernel, instead of Gaussian functions, which is more adequate to the geometry of the CGR domain.

The Entropic Profiles proposed here assess point/symbol normalized deviations from a mean composition signature. EP calculation was based on a density estimation value per position, thus depicting local sequence information related with the statistical significance of a motif, measured as its global over- or under-representation. Furthermore, it was shown that using this kernel the EP can be calculated independently from a particular representation. The local genome scale (or resolution) is defined by the combination of parameters for which a particular suffix emerges. Therefore, this scanning procedure identifies simultaneously the position and the scale at with the sequence composition is singular, by focusing and adjusting the best parameters locally and then looking back to the overall sequence. There is a strong biological rationale for such an approach as the genome is organized to conserve motifs at different scales (lengths) and with varying stringency. The underlying hypothesis is that over- or underrepresented motifs may be indicative of important biological functions.

This conclusion was illustrated with the analysis of artificial DNA sequences, reference genomic datasets and also whole genomes from *E. coli *and *H. influenzae*, where known regulatory components and motifs were correctly recovered – both as regards position and scale (length) of the conserved segments. By spanning the parameter space of this new function it was possible to study the local scale for which a given suffix and position were implicit. This effort highlighted the interaction between several methodologies in this field. Specifically, it greatly simplifies the exploration of fundamental relationships between distinct sequence analysis approaches and concepts such as metrics on strings, information theory and entropy, iterated function systems and statistical significance of DNA segments, providing a common ground in kernel-based learning theory.

The procedure proposed here is easily extendable to other kernel function classes, which might be more adequate to model specific traits or genomes. Future work includes the generalization for point mutations and also dealing with nested or embedded motifs.

The proposed entropic profiles provide promising new tools for the study of biological sequences, allowing the quantification of repeatability and identifying key parameters for which relevant features arise.

## Methods

This section recalls the background work that led to the new analysis described here and defines the main concepts proposed, namely: the CGR/USM representation of DNA sequences; the assessment of entropy in biological sequences and definition of local Entropic Profile (EP); the use of specialized kernel density estimation functions and its conjugation with the EP method.

### CGR/USM representation of DNA sequences and Parzen's method

The CGR/USM representation, introduced in [6] and generalized to higher-order alphabets in [41], allows the mapping of a discrete DNA sequence onto ℝ^*n*^. Formally, the CGR mapping *x*_*i *_∈ ℝ^2 ^of a *N*-length DNA sequence **S **= *s*_1 _*s*_2 _... *s*_*N*_, *s*_*i *_∈ A= = {*A, C, G, T *}, *i *= 1, ..., *N *is given by Equation 1:

$$\{\begin{array}{cc} \begin{array}{l} x_{0}=\left(0.5,0.5\right)\\ x_{i}=x_{i-1}+\frac{1}{2}\left(y_{i}-x_{i-1}\right),i=1,...,N \end{array} & \text{where }y_{i}=\{\begin{array}{ccc} \left(0,0\right) & \text{if} & s_{i}='A'\\ \left(0,1\right) & \text{if} & s_{i}='C'\\ \left(1,0\right) & \text{if} & s_{i}='G'\\ \left(1,1\right) & \text{if} & s_{i}='T' \end{array} \end{array}$$

The properties and generalizations of this method have already been studied and extensively applied as a consequence to the natural development of alignment-free techniques for sequence comparison [13,42].

As previously, the variables employed in this work will be the USM coordinates sample points {*x*_*i*_}_*i *= 1, ..., *N *_that correspond to the symbols {*s*_*i*_}_*i *= 1, ..., *N *_in the original sequence.

In particular, it was seen in the previous report that these points could be adequately used to estimate the Rényi entropy of the original sequence through the Parzen's window density estimation method [43]. This is a non-parametric technique used to estimate a probability density function *f *from a sample. This method is one of the most widely used kernel-based methods and consists on the choice of a weighting function or kernel *κ*_*θ *_(*x*). The estimation $\hat{f}$ (*x*) of a random vector *x *is a linear combination of the kernels centered in the observed sample points *a*_*i*_, *i *= 1, ..., *N*, and is defined for a specific window width *τ *(Eq.2):

$$\hat{f}\left(x;a,\theta \right)={\hat{f}}_{\theta }\left(x;a\right)=\frac{1}{N\tau }\sum_{i=1}^{N}\kappa_{\theta }\left(\frac{x-a_{i}}{\tau }\right)$$

In that former report [19] Gaussian or normal distribution functions were used in order to estimate the Rényi quadratic entropy of the CGR of a given DNA sequence. Due to important algebraic simplifications and properties of the Gaussian kernel it was shown that this calculation was obtained by using a simple potential function of the CGR map.

### Entropic profile definition

The former equations provide a natural method to extract local information from a DNA sequence. By calculating the values $\hat{f}$_*θ *_(*x*_*i*_) for each coordinate *x*_*i *_that represents the *i*^th ^symbol in the original sequence and parameter set *θ*, it is possible to plot, for each position *i *= 1, ..., *N*, normalized values $\hat{g}$_*θ *_(*x*) ≡ $\hat{g}$_*θ *_(*x*; *a*) of the density function estimated previously, obtained as the number of standard deviations from the mean (taking into account all the sample points or symbols, omitted for notation simplification):

$${\hat{g}}_{\theta }\left(x\right)\equiv \frac{{\hat{f}}_{\theta }\left(x\right)-m_{\theta }}{s_{\theta }},\text{ with }m_{\theta }=\frac{1}{N}\sum_{i=1}^{N}{\hat{f}}_{\theta }\left(x_{i}\right)\text{ and }s_{\theta }=\sqrt{\frac{1}{N-1}\sum_{i=1}^{N}{\left({\hat{f}}_{\theta }\left(x_{i}\right)-m_{\theta }\right)}^{2}}$$

In fact, this corresponds to extracting the local density, estimated for each coordinate that represents a symbol in the original sequence context. For example, if a particular motif appears more often than what would be expected by chance, the density estimation for that particular position/coordinate will be higher than the average *m*_*θ*_.

For each parameter set *θ *one can define the *Entropic Profile EP*_*θ *_(*i*) ≡ $\hat{g}$_*θ *_(*x*_*i*_), *i *= 1, ..., *N*, that measures precisely the density deviations from the mean in each coordinate, or equivalently, in each last symbol of all the suffixes appearing in the original sequence.

Therefore, these values obtained with the kernel estimations are related to the statistical significance of the corresponding suffix present at that particular position, since they represent a density, which is strongly associated with the degree of repetition of a given suffix in the sequence.

It is worth noting that the proposed entropic profiles are a descriptive measure of local DNA properties and that a full extensive comparison with other methods that search for motifs and assign *p*-values to the results are out of the scope of this work. Future efforts will quantitatively compare these profiles with other models, e.g. Markov chain models, to confirm for the quantitative correspondence between methods on the assessment of under and over-representation of motifs.

### Fractal kernel definition

The former approach used Gaussian distribution function to model the generalized Markov models. One possible drawback of this methodology is related with the domain issue above mentioned, since the normal distribution function is defined in ℝ^*n *^whereas the CGR/USM domain is explicitly defined in unit hypercubes. This concern lead to the development of another kernel [23] to be used in the CGR density estimation, which is recalled, reformulated and further discussed in this section.

Let *χ*_*A *_: *X *→ {0,1}, *A *⊂ *X *⊆ ℝ, be an indicator or characteristic function such that:

$$\chi_{A}\left(x\right)=\{\begin{array}{cc} 1 & \text{if }x\in A\\ 0 & \text{if }x\notin A \end{array}$$

Each function $I{'}_{k,x_{j}}$ : *X *→ {0,1} with parameters *k *and *x*_*j *_is defined for a point *x *∈ *X *as:

$$I{'}_{k,x_{j}}\left(x\right)=\chi_{A_{k,x_{j}}}\left(x\right)$$

where the interval $A_{k,x_{j}}$ depends on the point *x*_*j *_∈ *X *and on the resolution *k *chosen:

$$A_{k,x_{j}}=\left(2^{-k}\left\lfloor 2^{k}x_{j}\right\rfloor ,2^{-k}\left\lfloor 2^{k}x_{j}\right\rfloor +2^{-k}\right)$$

and ⌊*x*_*j*_⌋ denotes the *floor *function. The interval above defined $A_{k,x_{j}}$ has length *V *($A_{k,x_{j}}$) = 2^-*k*^.

Intuitively, this function rounds the value of *x*_*j*_, respecting the borders of the regions that represent specific *k*-tuples, which are always given by multiples of 2^-*k *^(see figure 1 in[19]). This might also be interpreted as the number of common digits of the binary representation of *x*_*j *_and *x*, up to the *k*^*th *^decimal digit. This is more clearly deduced using numeric representation in base 2.

For the CGR mapping $\vec{x}$ ≡ (*x*^(1)^, *x*^(2)^) ∈ ℝ^2^, the 2D step function for a point ${\vec{x}}_{j}\equiv \left(x_{j}^{\left(1\right)},x_{j}^{\left(2\right)}\right)$ ∈ ℝ^2 ^is defined as $I_{k,{\vec{x}}_{j}}\left(x^{\left(1\right)},x^{\left(2\right)}\right)=I{'}_{k,x_{j}^{\left(1\right)}}\left(x^{\left(1\right)}\right)\times I{'}_{k,x_{j}^{\left(2\right)}}\left(x^{\left(2\right)}\right)$, i.e., the function is 1 when both coordinates *x*^*(1) *^and *x*^*(2) *^belong the above mentioned intervals and is zero otherwise. This is due to the indicator function property *χ*_*A *∩ *B *_= *χ*_*A *_*χ*_*B*_. For sake of clarity and notation simplification, in the following formulas all the variables *x *and *x*_*j *_will be assumed in ℝ^2 ^otherwise stated, i.e. $I_{k,{\vec{x}}_{j}}\left(x^{\left(1\right)},x^{\left(2\right)}\right)\equiv I_{k,x_{j}}\left(x\right)$.

The kernel *κ*^*f *^(*x*) used in this work and extensively presented in [23] is based on the linear combination of block functions *I*_*k*_, using particular resolutions *k *and a parameter *h *that defines the height (or weight) of each block:

$$\kappa^{f}\left(x\right)\equiv \kappa_{L,x_{j}}\left(x\right)=\sum_{k=0}^{L}h_{k}\cdot I_{k,x_{j}}\left(x\right).$$

Additionally, considering the restriction of probability density functions, the following equation is obtained:

$$\int \kappa_{L,x_{j}}\left(x\right)\text{d}x=1\Rightarrow \sum_{k=0}^{L}h_{k}2^{-2k}=1$$

since $\int I_{k,x_{j}}\left(x\right)\text{d}x=V\left(A_{k,x_{j}}\right)=2^{-2k}$ and $V\left(A_{k,{\vec{x}}_{j}}\right)=V\left(A_{k,x_{j}^{\left(1\right)}}\right)V\left(A_{k,x_{j}^{\left(2\right)}}\right)=2^{-2k}$.

Defining *φ *as the (constant) ratio between two consecutive volumes *A*_*k *_and *A*_*k*-1_, *k *= 1, ..., *L *(in 2D):

$$\varphi =\frac{V\left(A_{k}\right)}{V\left(A_{k-1}\right)}=\frac{1}{4}\frac{h_{k}}{h_{k-1}}\Rightarrow h_{k}=4\varphi \cdot h_{k-1}={\left(4\varphi \right)}^{k}\cdot h_{0},$$

it is possible to express this restriction in terms of *φ *as:

$$h_{0}\sum_{k=0}^{L}\varphi^{k}=1$$

And finally the (normalized) kernel $\kappa_{L,\varphi ,x_{j}}$ (*x*) with parameters *L*, *φ *and *x*_*j *_is:

$$\kappa_{L,\varphi ,x_{j}}\left(x\right)=\sum_{k=0}^{L}h_{k}\cdot I_{k,x_{j}}\left(x\right)=\frac{\sum_{k=0}^{L}{\left(4\varphi \right)}^{k}\cdot I_{k,x_{j}}\left(x\right)}{\sum_{k=0}^{L}\varphi^{k}}$$

**Figure 8:**
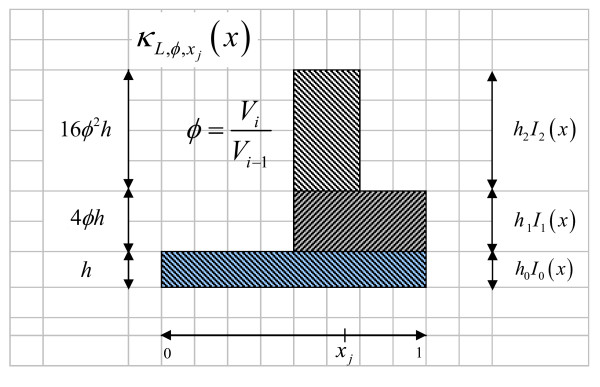
**Example of the proposed fractal kernel *κ*(*x*)**. Fractal kernel construction projected to one-dimension, for *L *= 2 and arbitrary *φ*.

The underlying idea is to weight, by powers of 4 *φ*, each step function $I_{k,x_{j}}$ (*x*), which corresponds to a sort of generalized Markov model. An illustration of this kernel function (projected to one-dimensional space) is given in Figure 8 for *L *= 2 which correspond to three blocks $I_{k,x_{j}}$ (*x*), *k *= 0,1,2.

Another important property of this function *κ *is its symmetry regarding *x*_*i *_and *x*_*j*_, in fact, $\kappa_{L,\varphi ,x_{j}}\left(x_{i}\right)=\kappa_{L,\varphi ,x_{i}}\left(x_{j}\right)$ since ${I^{\prime }}_{k,x_{i}}\left(x_{j}\right)={I^{\prime }}_{k,x_{j}}\left(x_{i}\right)$. Actually, if *x*_*i *_belongs to the interval *A*_*k *_means that *x*_*i *_and *x*_*j *_have the same binary expansion up to the *k *digit, which obviously implies symmetry.

This allows a straightforward generalization under kernel learning theory in which specific transformation of the data with kernel functions induce dot products and norms in other function spaces [44]. In fact, this kernel is related with the Cantor distance in strings, which measures precisely the suffix similarity.

Furthermore, it should be clear that this new fractal kernel is more adjusted to the CGR geometry: instead of Gaussian functions that span all ℝ^*n *^domain the proposed *κ *(*x*) is defined on unit hypercubes, which is definitely more in agreement with these iterative maps.

### Entropic profiles with fractal kernels

When using the above-defined fractal-based kernel, the expression for the estimation for the entropic profile is significantly simplified, thus allowing its optimal and straightforward calculation. In fact, for a particular coordinate, each density block is only different from zero if the points in that neighborhood are close, in the sub-quadrant sense. In other words, for one position, the only non-zero blocks of length *k *correspond to the nearest points, which are at a distance less than 2^-*k *^apart.

Another important note is that this particular kernel, contrary to the Gaussian which only has two parameters (mean and variance), depends on the point *x*_*j*_: in effect, the format of the kernel varies according to the rounding procedure and the particular coordinate *x*_*j *_considered.

Therefore, the Parzen density estimation for position *i *or point *x*_*i *_is given as a function of all the other sample coordinates *x*_*j*_, *j *= 1, ..., *N*, and parameter set *θ *≡ {*L*, *φ*}, where *L *represents the Markov resolution and *φ *is a smoothing parameter:

$${\hat{f}}_{L,\varphi }\left(x_{i}\right)=\frac{1}{N}\sum_{j=1}^{N}\kappa_{L,\varphi ,x_{j}}\left(x_{i}\right)$$

By simple algebraic simplification and using Eq.4 one obtains a more condensed formula:

$${\hat{f}}_{L,\varphi }\left(x_{i}\right)=\frac{1}{N\sum_{k=0}^{L}\varphi^{k}}\sum_{k=0}^{L}{\left(4\varphi \right)}^{k}\sum_{j=1}^{N}I_{k,x_{j}}\left(x_{i}\right)\text{ where}$$

$$I_{k,x_{j}}\left(x_{i}\right)=\{\begin{array}{ll} 1 & \text{if }x_{i}^{\left(1\right)}\in A_{k,x_{j}^{\left(1\right)}}\text{ and }x_{i}^{\left(2\right)}\in A_{k,x_{j}^{\left(2\right)}}\\ 0 & \text{otherwise} \end{array}$$

Due to the CGR suffix property, the last condition is equivalent of having the same suffix of length *k*, i.e., $x_{i}^{\left(1\right)}\in A_{k,x_{j}^{\left(1\right)}}$ and $x_{i}^{\left(2\right)}\in A_{k,x_{j}^{\left(2\right)}}$ if and only if the string with length *k *corresponding to the CGR coordinate *x*_*i *_is the same as the one represented by coordinate *x*_*j*_. Therefore the sum $\sum_{j=1}^{N}I_{k,x_{j}}$ (*x*_*i*_) that appears in the last equation is calculated by simply counting the number of common suffixes of length *k *shared through all the sequence ***S***:

$$\sum_{j=1}^{N}I_{k,x_{j}}\left(x_{i}\right)=\sum_{j=1}^{N}\delta_{s_{i}^{k}s_{j}^{k}}=count_{s}\left(s_{i-k+1}\cdots s_{i}\right)=c\left(\left[i-k+1,i\right]\right)$$

where *δ*_*ij *_is the Kronecker delta and $s_{i}^{k}$ is the suffix of length *k *that ends in position *i*.

Finally, and using this result, the Parzen density estimation with this kernel can be simplified to the formula given by the following Equation 5:

$${\hat{f}}_{L,\varphi }\left(x_{i}\right)=\frac{1+\frac{1}{N}\sum_{k=1}^{L}4^{k}\varphi^{k}\cdot c\left(\left[i-k+1,i\right]\right)}{\sum_{k=0}^{L}\varphi^{k}},L\geq 1$$

Computationally, this is a significant result since it allows the simplification of $\hat{f}$_*L*, *φ *_(*x*_*i*_): instead of having to calculate individual kernel function for each point and sum all the contributions, one can simplify the calculation up to a desired resolution or memory length *L*, greatly reducing the associated algorithmic complexity from quadratic to linear on *L *and sequence length. In the supplementary MATLAB functions available along this report this simplification was taken into account. In practice this is an important result since low resolutions *L *are commonly used, remembering that they represent Markov orders. Indeed, most approaches in sequence modeling use Markov orders below 8, which greatly simplifies the calculation time. Some limiting properties of the estimation *f *for different *φ *include:

$$\begin{array}{l} \underset{\varphi \to \infty }{\lim}{\hat{f}}_{L,\varphi }\left(x_{i}\right)=\frac{4^{L}}{N}\cdot c\left(\left[i-L+1,i\right]\right)=\frac{4^{L}}{N}\cdot c\left(L\text{-tuple suffix }i\right)\\ \underset{\varphi \to 0}{\lim}{\hat{f}}_{L,\varphi }\left(x_{i}\right)=1 \end{array}$$

These results show that the parameter *φ *is weighting different Markov chain models: *φ *= 0 means that a zero order, background (equal) frequencies are taken, whereas *φ *→ ∞ corresponds to weighting higher *L*-tuples, ignoring the lower order counts, which, in the limiting case, is equivalent to a *L*-order Markov chain.

In effect, $\hat{f}$_*L*, *φ *_(*x*_*i*_) can be interpreted as a linear combination of suffixes counts up to a given memory length, with increasing (*φ *> 1/4) or decreasing weights (*φ *< 1/4). These results came up as quite unpredictably, since the kernel defined above was based on a different rationale. It turned out that both perspectives are equivalent in terms of final formulation. It is also noteworthy the relation between this methodology and generalized Markov models and interpolated Markov chains (IMM). In fact, similar profiles were obtained recently [39] representing the shortest unique substrings in sequences.

In the application section when calculating the normalized values *EP*_*θ *_(*i*) ≡ $\hat{g}$_*θ *_(*x*_*i*_), one has to consider a burnt-in period corresponding to the first symbols in the sequence. Since the estimation of the profile is biased in the sense that only higher order tuples are considered, it is necessary to exclude these first points *f*(*x*_*i*_), *i *= 1, ..., *b*_*0*_, given that no information is provided for higher suffixes up to that position. For that reason, this correction was taken into account when using the EP normalized values. This border effect is nevertheless negligible and can be ignored for longer sequences. The background just presented will allow the representation of the entropic profiles *EP*_*θ *_≡ $\hat{g}$_*L*, *θ *_as a function of both *L *and *θ *and search for key parameters combinations to unravel the scale upon which important features might arise in the original DNA sequence.

### Markov Chain-based *p*-value calculation

In order to compare our method with previous efforts, we also report the p-values and respective statistical z-scores for the motifs analyzed. These values were calculated using first-order Markov Chain transition probability tables estimated directly from the whole sequences. This estimation was based on the relative frequency of each oligonucleotide, using pseudo-counts to avoid zero transition probabilities when necessary. After this step, the probability of each motif can be easily accessed along with their expected number of occurrences in a specific sequence. The calculation of the p-value of a motif *m *is therefore the probability of observing more counts N(*m*) than those expected under that given model, i.e., prob{N(*m*) ≥ N_obs_(*m*)}. The normal distribution was used as an approximation for the distribution of N(*m*), with expected values and variances described in [1]. These variances took into account the overlap capacity or period of each motif, as described in the same reference. Other approximations, such as using the Poisson distribution, give the same relative order for the motifs. The p-values calculated are reported for each motif referred in the text. To complement the analysis and since many of the motifs studied exhibit very low p-values, practically equal to zero, i.e. they are exceptionally frequent, the z-scores and their relative rank order was also reported. In this way a more accurate comparison can be performed.

## Competing interests

The author(s) declares that there are no competing interests.

## Authors' contributions

SV devised and developed the methodology, carried out the analysis and wrote the manuscript. JSA participated in the implementation of the algorithms, the design of the study and contributed to the analysis and interpretation of results. All authors read and approved the final manuscript.
